# Validation of a Simplified Tissue-to-Reference Ratio Measurement Using SUVR to Assess Synaptic Density Alterations in Alzheimer Disease with [^11^C]UCB-J PET

**DOI:** 10.2967/jnumed.124.267419

**Published:** 2024-11

**Authors:** Juan J. Young, Ryan S. O’Dell, Mika Naganawa, Takuya Toyonaga, Ming-Kai Chen, Nabeel B. Nabulsi, Yiyun Huang, Emma Cooper, Alyssa Miller, Jessica Lam, Kara Bates, Audrey Ruan, Kimberly Nelsen, Elaheh Salardini, Richard E. Carson, Christopher H. van Dyck, Adam P. Mecca

**Affiliations:** 1 Alzheimer’s Disease Research Unit, Yale School of Medicine, New Haven, Connecticut;; 2 Department of Psychiatry, Yale School of Medicine, New Haven, Connecticut;; 3 VA Connecticut Healthcare System, West Haven, Connecticut;; 4 Department of Radiology and Biomedical Imaging, Yale School of Medicine, New Haven, Connecticut;; 5 Department of Neuroscience, Yale School of Medicine, New Haven, Connecticut; and; 6 Department of Neurology, Yale School of Medicine, New Haven, Connecticut

**Keywords:** [^11^C]UCB-J, synaptic density, SUVR, PET, Alzheimer disease

## Abstract

Simplified methods of acquisition and quantification would facilitate the use of synaptic density imaging in multicenter and longitudinal studies of Alzheimer disease (AD). We validated a simplified tissue-to-reference ratio method using SUV ratios (SUVRs) for estimating synaptic density with [^11^C]UCB-J PET. **Methods:** Participants included 31 older adults with AD and 16 with normal cognition. The distribution volume ratio (DVR) using simplified reference tissue model 2 was compared with SUVR at short scan windows using a whole-cerebellum reference region. **Results:** Synaptic density was reduced in AD participants using DVR or SUVR. SUVR using later scan windows (60–90 or 70–90 min) was minimally biased, with the strongest correlation with DVR. Effect sizes using SUVR at these late time windows were minimally reduced compared with effect sizes with DVR. **Conclusion:** A simplified tissue-to-reference method may be useful for multicenter and longitudinal studies seeking to measure synaptic density in AD.

## Visual Abstract

**Figure:**
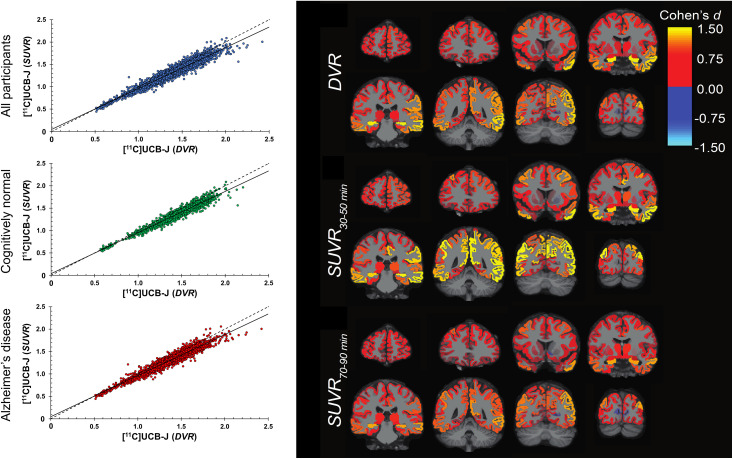

Alzheimer Disease (AD) pathology is traditionally characterized by the accumulation of extracellular amyloid-β plaques and intracellular neurofibrillary tangles. An equally important pathology, synaptic loss, is an early event in the disease process and a major structural correlate of cognitive impairment (1–3). PET imaging of synaptic vesicle glycoprotein 2A has emerged as a novel biomarker of synaptic density in AD (4*,*^5^). Using (*R*)-1-((3-(^11^C-methyl-^11^C)pyridin-4-yl)methyl)-4-(3,4,5-trifluorophenyl)pyrrolidin-2-one ([^11^C]UCB-J) PET, we and others have demonstrated widespread gray matter synaptic loss (6), which correlated with cognitive performance (7*,*^8^) and tau deposition (9–12). Our past analyses have used an outcome of distribution volume ratio (DVR) from simplified reference tissue model 2 kinetic modeling. Simplified methods of image acquisition and quantification would facilitate multicenter and longitudinal studies. Thus, the objective of this study was to validate a simplified tissue-to-reference ratio method using SUV ratios (SUVRs).

## MATERIALS AND METHODS

### Participants

A subset of previously reported participants (6) who had full 90-min scans were included in this analysis. Participants 55–85 y of age were recruited and assessed either to be cognitively normal (CN) or to meet the diagnostic criteria for amnestic mild cognitive impairment (13) or dementia due to probable AD (14). Via [^11^C]Pittsburgh compound B PET, mild cognitive impairment and dementia participants were amyloid-β–positive and CN participants were amyloid-β–negative (6*,*^15^*,*^16^). The study protocol was approved by the Yale Human Investigation Committee. All participants gave informed consent.

### Acquisition of Data

[^11^C]UCB-J PET images were acquired as previously described (17). Participants underwent 90-min PET scans on a High Resolution Research Tomograph (Siemens Medical Solutions) after a bolus injection of [^11^C]UCB-J (553 ± 199 MBq) over 1 min. Dynamic scan data were reconstructed in 27 frames (6 × 0.5 min, 3 × 1 min, 2 × 2 min, 16 × 5 min) with corrections for attenuation, normalization, scatter, randoms, and dead time using the MOLAR algorithm (motion-compensation ordered subset expectation maximization list-mode algorithm for resolution-recovery reconstruction) (18). Motion was corrected using the Polaris Vicra sensor (NDI Systems) (19). T1-weighted MRI was used for exclusion of structural abnormalities and for coregistration with PET scans. Parametric images of DVR were generated using simplified reference tissue model 2 (0–60 min) with a whole-cerebellum reference region (6*,*^15^*,*^16^). SUVR images were generated by summing frames across 20- and 30-min windows from 30 to 90 min and normalized to the whole cerebellum. Regions of interest were defined using the FreeSurfer (version 6.0) segmentation and parcellation pipeline (20).

### Simulation

A simulation study was conducted to examine the effect of perfusion differences on group-level comparisons of SUVR and Cohen *d* within AD and CN groups across various time points. This simulation was based on data from our previous study (4) using a 1-tissue-compartment model to estimate parameters for *K*_1_ (perfusion) and *k*_2_ (tracer efflux) in both the hippocampus and the cerebellum for each participant (Supplemental Table 1; supplemental materials are available at http://jnm.snmjournals.org). Effects of perfusion differences were explored by simulating hippocampal time–activity curves for each individual with AD according to varying *K*_1_ percentage differences (0%, 10%, 20%, 30%, and 40%) relative to the average *K*_1_ of the CN group; these correspond to *R*_1_ (relative tracer delivery) group percentage differences of 3%, 13%, 22%, 32%, and 42%, respectively. Percentage differences in mean SUVR between CN and AD groups and Cohen *d* values were computed at each time point and were compared with the percentage differences in DVR for all *K*_1_ settings. The supplemental methods provide more detailed information.

### Statistical Analysis

Group differences were evaluated using χ^2^ tests for categoric variables and unpaired *t* tests for continuous variables. Linear regression was used to assess the associations between SUVRs and DVR (via kinetic modeling). The effect size (Cohen *d*) to determine the group difference (CN vs. AD) was calculated for each PET outcome. Linear regression was used to assess the correlation between effect sizes using DVR and SUVR. Regression equations and Pearson correlation coefficients (*r*) were reported for each regression model. Two-tailed *P* values were reported. Statistical analyses were performed using SPSS 21.0 (IBM Corp.).

## RESULTS

Participant characteristics are shown in Table 1.

**TABLE 1.** Participant Characteristics

Characteristic	CN	AD	*P*
Participants (*n*)	16	31 (9 mild cognitive impairment; 22 mild dementia)	
Sex (*n*)			0.76
Male	7	15	
Female	9	16	
Age (y)	70.6 ± 7.7 (60.8–82.7)	69.8 ± 8.0 (50.3–84.5)	0.73
Education (y)	17.8 ± 2.20 (12–20)	16.2 ± 2.39 (12–20)	0.03
CDR, global	0 ± 0 (0–0)	0.77 ± 0.25 (0.5–1.0)	<0.01
MMSE	29.3 ± 1.2 (27–30)	22.9 ± 3.7 (14–30)	<0.01
*APOE* ɛ4 copy number (*n*)			<0.01
0	14 (87.5%)	9 (29.0%)	
1	2 (12.5%)	14 (45.2%)	
2	0 (0%)	8 (28.8%)	

CDR = clinical dementia rating; MMSE = mini-mental state examination.

Categorical data are counts; continuous data are mean ± SD followed by range in parentheses.

**FIGURE 1.**
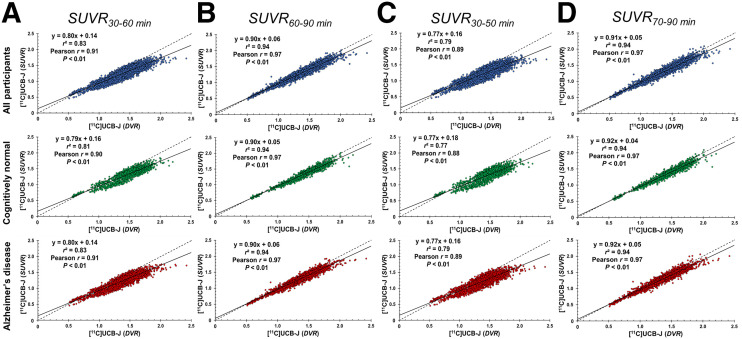
Correlations between DVR and SUVR at 30–60 min (A), 60–90 min (B), 30–50 min (C), and 70–90 min (D) for all participants and for CN and AD groups in all regions of interest.

DVR correlated strongly with SUVR across all brain regions and within each region (Fig. 1; Supplemental Fig. 1; Supplemental Table 2). The correlation was strongest for the 60- to 90-min and 70- to 90-min scan windows and weakest for the 30- to 50-min window. A similar pattern was seen in all participants or when CN or AD participants were analyzed separately. A comparison of regression lines to the line of identity showed that SUVR was less biased at later scan windows than at earlier scan windows (Fig. 1).

**FIGURE 2.**
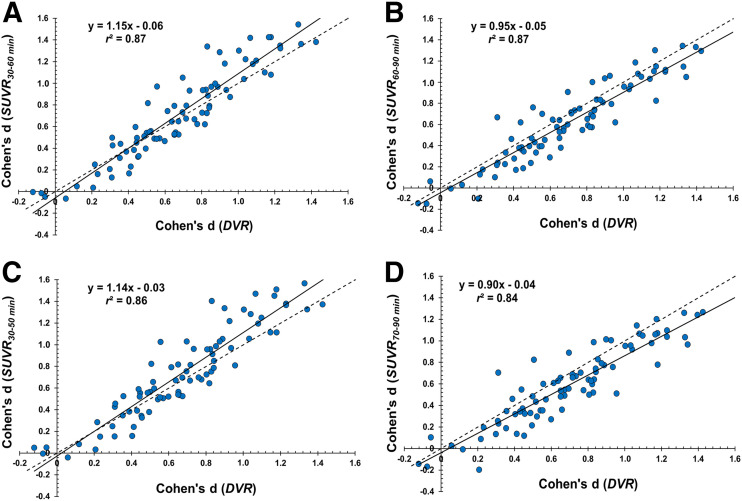
Correlations between effect sizes (Cohen *d*) in all regions of interest as measured by DVR and SUVR at 30–60 min (A), 60–90 min (B), 30–50 min (C), and 70–90 min (D).

**FIGURE 3.**
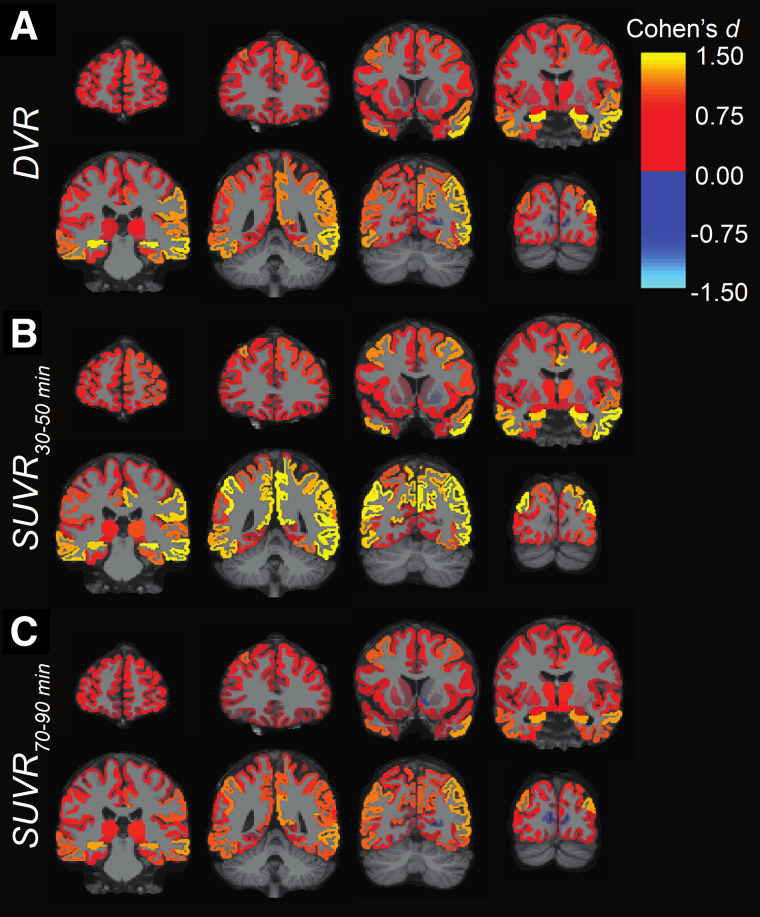
Effect size (Cohen *d*) maps of DVR (A), SUVR at 30–50 min (B), and SUVR at 70–90 min (C) for comparison between CN and AD groups. Effect size statistics are displayed for all analyzed regions and overlaid on MNI template T1-weighted MR images.

The effect sizes for the difference between CN and AD groups using SUVR were larger during earlier scan windows than during later scan windows, with the largest effect size being calculated for the 30- to 50-min scan window (Cohen *d* = 0.78) and the smallest effect being calculated for the 70- to 90-min window (Cohen *d* = 0.6) (Table 2). The effect size for SUVR at each scan window correlated strongly with the effect size for DVR (Table 2; Fig. 2). Early scan windows tended to overestimate the effect size, and later scan windows tended to underestimate the effect size, compared with DVR (Fig. 2). For specific regions, the same pattern of decreasing effect size at later time windows was consistently seen in the entorhinal cortex, lateral temporal cortex, prefrontal cortex, posterior cingulum/precuneus, lateral parietal cortex, lateral occipital cortex, and medial occipital cortex (Fig. 3; Supplemental Fig. 2).

**TABLE 2.** Correlation of Effect Sizes to Detect Differences in Synaptic Density Between AD and CN Groups

Parameter	Pearson *r*	*P*	Cohen *d* (mean ± SD)
SUVR window			
30–60 min	0.93	<0.01	0.75 ± 0.45
40–70 min	0.94	<0.01	0.70 ± 0.43
50–80 min	0.94	<0.01	0.66 ± 0.40
60–90 min	0.93	<0.01	0.63 ± 0.37
30–50 min	0.93	<0.01	0.78 ± 0.45
40–60 min	0.91	<0.01	0.72 ± 0.44
50–70 min	0.94	<0.01	0.67 ± 0.42
60–80 min	0.94	<0.01	0.64 ± 0.38
70–90 min	0.91	<0.01	0.60 ± 0.36
DVR	—	—	0.71 ± 0.36

Pearson *r* is for correlation between effect sizes (Cohen *d*) derived from DVR vs. each SUVR scan window.

**FIGURE 4.**
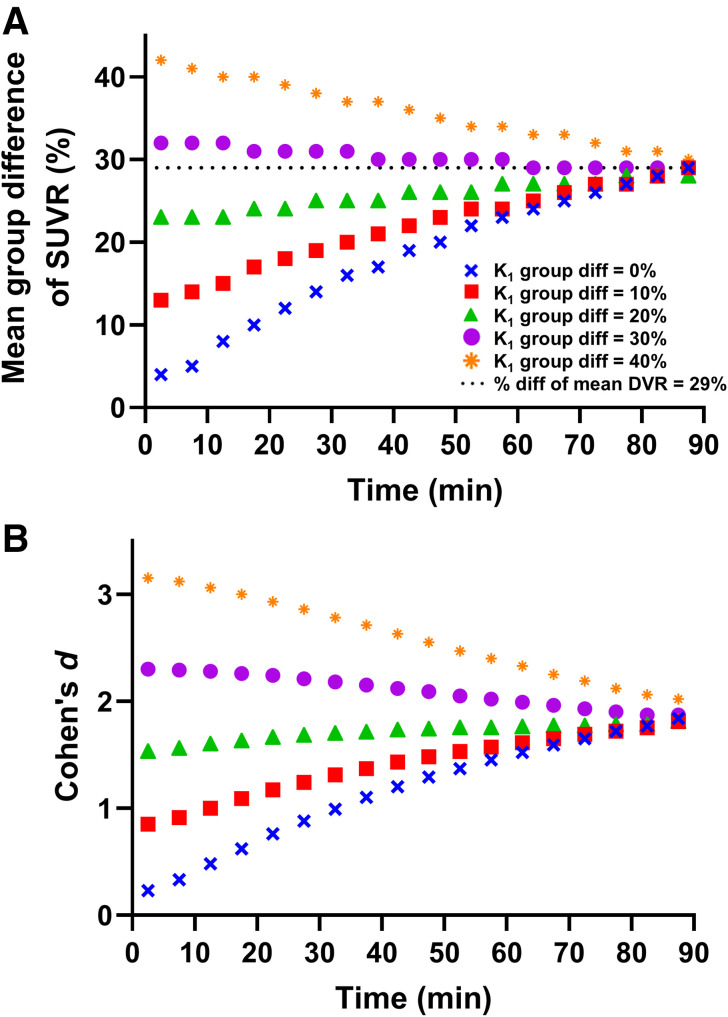
Simulation of group difference in mean SUVR (A) and Cohen *d* (B) for various perfusion changes.

The simulation model demonstrates how the percentage difference in SUVR (AD vs. CN) changes during the scan time (Fig. 4). At early scan times, the percentage difference approaches the difference in relative perfusion. As scan time increases, the percentage difference approaches the true difference using mean DVR. Thus, SUVR calculated from earlier scan windows reflects differences in perfusion, and SUVR calculated from later windows reflects specific tracer binding (synaptic density).

## DISCUSSION

SUVR calculated with later scan windows (60–90 or 70–90 min) had the strongest correlations with the previously validated method of calculating DVR with simplified reference tissue model 2 and a whole-cerebellum reference region (6*,*^8^).

SUVR generated using earlier scan windows of 30–60 or 30–50 min demonstrated the largest effect sizes to detect differences between AD and CN groups compared with later scan windows and DVR (Table 2). There was some region specificity as seen in early AD–affected regions such as the hippocampus, where no SUVR scan window demonstrated a larger effect size than DVR (Fig. 3; Supplemental Fig. 2). Correlation analyses between SUVR and DVR indicate that early scan windows may overestimate, and later scan windows may underestimate, group differences (Fig. 2; Supplemental Fig. 2). Our simulation suggests that SUVR from earlier time frames represents group differences in both synaptic density and perfusion/metabolism (Fig. 4). This is compatible with our previous [^18^F]FDG PET and [^11^C]UCB-J study, in which group differences in metabolism were generally larger than those in synaptic density (21).

This study using the whole cerebellum as a reference region expands on the validation of a simplified quantification method for [^11^C]UCB-J that used the centrum semiovale as a reference region (22). We previously reported that whole cerebellum is the preferred reference region in AD because the disease-related difference in distribution volume was nonsignificant and lower than for the centrum semiovale and because DVR using the cerebellum yielded significantly lower coefficients of variation when using 1-tissue-compartment modeling or the simplified reference tissue model 2 (6).

## CONCLUSION

This study supports the use of SUVR with a whole-cerebellum reference region calculated during 60- to 90-min or 70- to 90-min scan windows for [^11^C]UCB-J–based investigations of AD. SUVRs at late time windows are minimally biased and highly correlated with DVR while having minimal bias in effect size compared with DVR.

## DISCLOSURE

Support was received from P50AG047270, K23AG057784, R01AG052560, R01AG062276, and T32AG1934. No other potential conflict of interest relevant to this article was reported.

## Notes

KEY POINTS **QUESTION:** Is SUVR a valid outcome for assessing synaptic density in AD with [^11^C]UCB-J? **PERTINENT FINDINGS:** SUVR calculated with 60- to 90-min or 70- to 90-min scan windows correlated strongly with DVR and was minimally biased. **IMPLICATIONS FOR PATIENT CARE:** SUVR can be used to quantify synaptic loss in AD.
